# Implantable biomedical materials for treatment of bone infection

**DOI:** 10.3389/fbioe.2023.1081446

**Published:** 2023-01-30

**Authors:** Wang Shuaishuai, Zhu Tongtong, Wang Dapeng, Zhang Mingran, Wang Xukai, Yu Yue, Dong Hengliang, Wu Guangzhi, Zhang Minglei

**Affiliations:** ^1^ Department of Orthopedics, China-Japan Union Hospital of Jilin University, Changchun, China; ^2^ Department of Orthopedics, Siping Central Hospital, Siping, China

**Keywords:** biological materials, bone infection, multifunctional material, implantable material, treatment of bone infection, progress of infection treatment, multifunctionalization of materials

## Abstract

The treatment of bone infections has always been difficult. The emergence of drug-resistant bacteria has led to a steady decline in the effectiveness of antibiotics. It is also especially important to fight bacterial infections while repairing bone defects and cleaning up dead bacteria to prevent biofilm formation. The development of biomedical materials has provided us with a research direction to address this issue. We aimed to review the current literature, and have summarized multifunctional antimicrobial materials that have long-lasting antimicrobial capabilities that promote angiogenesis, bone production, or “killing and releasing.” This review provides a comprehensive summary of the use of biomedical materials in the treatment of bone infections and a reference thereof, as well as encouragement to perform further research in this field.

## 1 Introduction

Bone infection, usually referring to that caused by bacterial infection in which bone lesions completely destroy the bone, is not common but it is a catastrophic condition (Porrino et al., 2020). According to statistics, when comparing the periods of 1969–1979 and 2000–2009, the incidence of orthopedic infections in the United States increased from 11.4 to 24.4 times per year per 100,000 residents, respectively (Kremers et al., 2015). In Spain, when comparing 1985–1991 to 2007–2011, the incidence of orthopedic infections increased from 2.34 to 5.78 per year per 100,000 inhabitants, respectively (Murillo et al., 2015). The overall prevalence of orthopedic infections in Germany increased from 15.5 to 16.7 cases per year per 100,000 inhabitants from 2008 to 2018 (Walter et al., 2021), and that in South Korea increased from 7.8 to 9.1 in 2008–2016 (Kim et al., 2019). It is expected that, in the near future, the number of orthopedic implant-related infections will further increase (Piuzzi et al., 2019).

Orthopedic implant-related infection is one of the major early postoperative complications in artificial joint surgery, usually occurring within 3 months after surgery. Osteomyelitis (OM) and prosthetic joint infection (PJI) are serious deep tissue infections. There are various sources of infection such as bacteremia, spread or injury from nearby tissues, and after surgery or foreign body implantation (Price et al., 2018; Scialla et al., 2021). Implant infection is most commonly caused by bacteria of coagulase negative Staphylococcus. *Staphylococcus aureus (S. aureus)* is often thought of as the main pathogen (Dapunt et al., 2016).

The treatment of PJI has traditionally used a 2-stage revision. Phase 1 revisions are now increasingly used (Aboltins et al., 2016). However, patients may require long-term antibiotic therapy and repeated revision surgery leads to dysfunction and even amputation. In addition to revision joint replacement surgery and other implant-related infections, such as open fractures, the surgical site of primary joint arthroplasty infection is more common and a difficult disease to cure clinically (Mills et al., 2016). Although systemic antibiotics are currently one of the most important and effective methods to treat bone infections, their use can lead to bacterial biofilm and bacterial resistance. For example, vancomycin is the gold standard in the treatment of methicillin-resistant *S. aureus* (MRSA) infection, but the minimum inhibitory concentration (MIC) of vancomycin in the treatment of MRSA is on the rise (Satola et al., 2011; Rossato et al., 2018; Aljohani et al., 2020).

Although traditional surgical treatment of bone infection quickly relieves symptoms, it may cause patients to become prone to repeated infections and severe surgical trauma. There is an urgent need to develop new treatment modalities to achieve better therapeutic effects. At present, the use of antibacterial materials for the treatment of bone infection has become a hot research topic. To reduce the morbidity of implant-related bacterial infections, biological medicine and the use of antibacterial materials is being given more attention (Ding et al., 2022).

Common antibacterial materials can be divided into organic, inorganic and a combination of any two of these materials. Organic material, such as polymethyl methacrylate (PMMA) bone cement mixed with antibiotics, has been widely used clinically in the past (Frank et al., 2011; Oei et al., 2012; Matos et al., 2015; Al Thaher et al., 2021). However, the polymerization temperature of PMMA is very high, which restrict the use of antibiotics (Nogueira et al., 2013). Therefore, more reliable antibacterial activity materials were developed. Inorganic materials (e.g., calcium sulfate, hydroxyapatite), which have received much attention in recent years, have begun to be widely used clinically (Qin et al., 2015a; McConoughey et al., 2015; Wang et al., 2018). Due to the biodegradable properties of these materials, they are gradually degraded within the body without secondary surgery, reducing repeated operations caused by trauma and other stimuli. Additionally, this reduces the amount of antibiotics used, which is accompanied by prolonged release, so that local antibiotics can be more effective. However, antimicrobial materials have limited applications, and often lack the functions we need, such as promoting osteogenesis, angiogenesis. As research and development of antibacterial materials has advanced, multifunctional implant antibacterial materials have become a trend. These materials combine biological compatibility and coagulation function, promote osteoblast proliferation, and possess multiple advantages of resistance to infection (Ding et al., 2018).

Although the combination of biomedical materials and antibiotics has been a huge success, implant-related infections continue to add pressure to the global healthcare system (Malizos, 2017). Due to the complex mechanism of bacterial infection and the barriers to delivery of antibiotics to the site of infection, the choice of antibiotic delivery methods has also attracted attention. Microstructural surface morphology changes in antimicrobial materials also enhance the functionality of the material (Rizzello et al., 2013; Hasan and Chatterjee, 2015; Jaggessar et al., 2017). Antibiotic delivery methods commonly used today are coatings, nanoparticles, three-dimensional (3D) scaffolds, and hydrogels (Gao et al., 2014; Nikolova and Chavali, 2019; Wei et al., 2019; Turner et al., 2021). The choice of appropriate antibiotic delivery enables us to deal with different kinds of bacterial infection in a more efficient manner.

**SCHEME 1:**
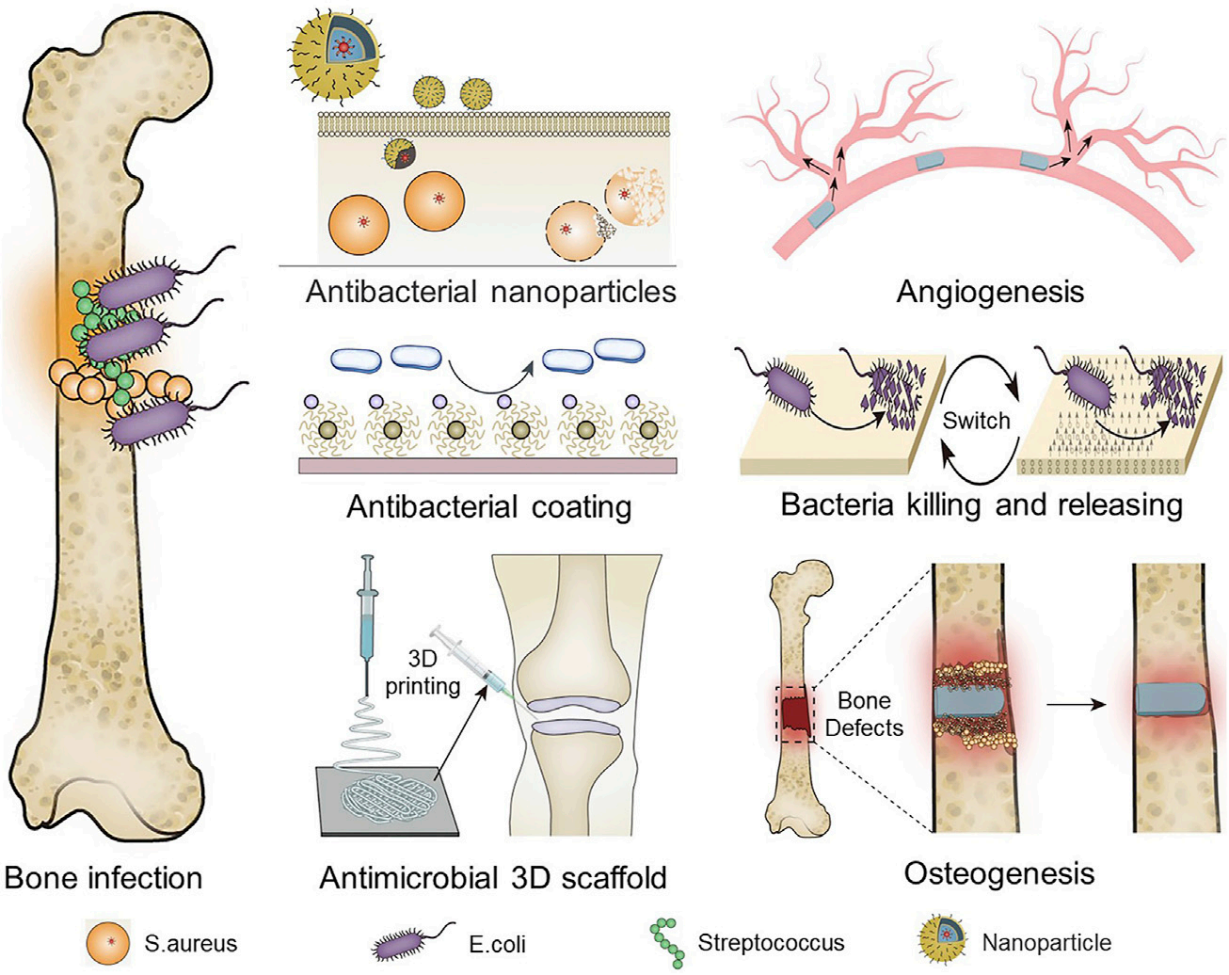
Antimicrobial treatment strategies for osteomyelitis.

We aimed to investigate the current research status of antibacterial materials, which are divided into organic materials, inorganic materials, and composite materials according to their chemical structures. We then propose that, with the current complexity of bone infection and the development of antibacterial materials, the introduction of multifunctional implantable antibacterial materials helps to overcome the difficulty in treating infections. Among these antibacterial materials, there are those that exhibit bactericidal or bacteriostatic activity by releasing antibiotics, those that repel bacterial adhesion, and those that combine multiple functions other than antibacterial properties, such as blood coagulation and promotion of osteoblast proliferation (Scheme 1). This review focuses on the characteristics of antibacterial materials in the treatment of orthopedic infections, the ability of antibacterial materials to load antibiotics, and the effect of drug sustained release on the prevention and treatment of orthopedic infections. We also focus on the application of antimicrobial materials for orthopedic-related infections to evaluate novel antimicrobial materials. Our analysis is intended to serve as a guide for researchers in the selection of appropriate antimicrobial materials for different conditions of orthopedic infection and to evaluate novel antimicrobial materials.

## 2 Application of antibacterial materials

Widely studied antimicrobial materials are divided into organic antimicrobial materials, inorganic antimicrobial materials, and organic-inorganic composites.

### 2.1 Organic materials

Organic materials contain synthetic organic polymer materials and natural organic materials. Among them, synthetic organic polymer materials include polymethyl methacrylate and polyglycolic acid, and natural organic materials include chitosan, alginate, and hyaluronic acid. They do not have antimicrobial properties *per se*, but function as carriers of drugs or antimicrobial materials.

#### 2.1.1 Synthetic organic polymer materials

##### 2.1.1.1 PMMA

PMMA-combined antibiotics have been used in the treatment of infected bone defects for many years in bone defects bearing a higher capacity after implantation. PMMA itself is not antibacterial, but it can be loaded with antibiotics, with the advantage of high concentrations of local antibiotics. However, polymerization temperatures are very high, and require that PMMA antibiotics have thermal stability (Lai et al., 2013). For this reason, gentamicin and tobramycin are often mixed into PMMA bone cement (Al Thaher et al., 2021); non-etheless, the heat released during the polymerization of PMMA still leads to the explosive release of antibiotics, resulting in a less-than-expected duration of effective antibiotic concentration. Therefore, to improve the efficiency of PMMA releasing antibiotics, the selection of different kinds of antibiotics and different preparation methods have become the focus of current research. Gentamicin-loaded silica nanoparticles are used to prolong gentamicin release for several weeks, and the gentamicin released from the antibiotic bone cement mixture has the same concentration as exhibited after 1 day. However, the subsequent release of antibiotics was less pronounced, with less gentamicin released after approximately 27 days (Al Thaher et al., 2018).

Chang et al. (2013) made different antibiotic loaded bone cements (ALBCs) containing vancomycin, teicoplanin, ceftazidime, imipenem, piperacillin, gentamicin, and tobramycin, respectively, and studied the antibacterial effects. According to their high-performance liquid chromatography results, all test samples on the first day showed a burst release. Then, in the next few days, the release rate dropped rapidly. The different ALBCs showed different release durations and additional daily release rates. Those containing gentamicin were more likely to have a longer release duration (10 days) than ALBCs containing ceftazidime (6 days), tobramycin (5 days), vancomycin, teicoplanin, imipenem, and piperacillin (all 2 days). The antibiotic concentrations of bone cement are also affected by the compressive ability, and the cellular compatibility with clindamycin (CLI) loaded PMMA cement had high compressive strength (∼120 MPa). Compared with the high loading of CLI cement, low loading CLI PMMA cement has better cell compatibility, because the CLI release rate is low, and cell adhesion on the surface of the cement is better (Pahlevanzadeh et al., 2021).

As an orthopedic implant material with a long history of use, PMMA bone cement has excellent shape plasticity and mechanical properties. However, when it polymerizes, it releases a large amount of heat, which may easily cause tissue damage and shorten drug release times. Furthermore, it is a biologically inert material, which has poor bonding with bone tissue and is prone to detachment. The solutions to these problems depend on the combined efforts of further studies.

##### 2.1.1.2 Poly lactic-co-glycolic-acid

Poly lactic-co-glycolic-acid (PLGA), a polylactic acid (PLA) and polyglycolic acid (PGA) copolymer, has undergone extensive research. PLGA is not antimicrobial, but it is widely used as a carrier in the treatment of bone infections. Due to its biocompatibility and biodegradability, PLGA degrades quicker, has adjustable mechanical performance (Houchin and Topp, 2009), has been shown to be an excellent delivery vehicle for antibiotics for the treatment of bone infections (Li et al., 2016; Ueng et al., 2016). In general, PLGA degradation and drug release rates are accelerated by greater hydrophilicity, increased chemical interactions between hydrolyzed groups, less crystallinity, and greater volume-to-surface ratio (Passerini and Craig, 2001). Garcia-Garcia et al. (2022) used a combination of vancomycin-loaded and daptomycin-loaded PLGA microspheres; the formulations retained their surrounding bone structure to a greater extent and cement modified with daptomycin-loaded PLGA microspheres may significantly preserve tissue structure from *S. aureus* infection.

Li et al. (2016) used PLGA mixed with vancomycin and hot compress molding to form an antibiotic bead to treat bone infection. An 18F-FDG PET scan was used to monitor responses to treatment of bone infection. The results showed successful eradication of *S. aureus* pathogens from the bone using the biodegradable PLGA vancomycin beads, with better cytocompatibility and osteogenic properties, and inhibition of biofilm formation. This is a promising treatment material for infectious bone defects. Furthermore, studies by gamma ray show more connections of hydrophilic iodide molecules to PLGA surfaces by poly (lactic-co-glycolic acid)-graft-polyvinylpyrrolidone/polyiodide (PLGA-g-PVP/I) (Wang et al., 2019a), and the antibacterial effects were verified by rat models. Scanning electron microscopy was performed to examine the morphology of the bacteria after incubation with these PLGA-g-PVP/I membranes. The results showed that the cells maintained their normal shape after 1 h of contact with the blank PLGA membrane. In contrast, cells were more severely damaged when incubated with PLGA-g-PVP/I membranes. The authors used two different incubation methods to verify the antimicrobial mechanism, and the results showed that the PLGA-g-PVP/I membrane in direct contact with bacteria was significantly superior in its leaching solution. *In vitro* glutathione (GSH) oxidation analysis of *S. aureus* after incubation with PLGA-g-PVP/I membranes for a period revealed that its antimicrobial properties were related to reactive oxygen species (ROS) production.

In sum, PLGA has good biocompatibility, biological safety, and biodegradability that makes it a widely used and effective drug carrier material. Antibiotics load by PLGA microspheres have potential advantages for the treatment of osteomyelitis (Ding and Zhu, 2018). However, the degradation conditions of PLGA are relatively harsh and need to be studied extensively.

#### 2.1.2 Natural organic polymer materials

Natural organic polymer materials have high biological activity, low toxicity, get easy degradability, rich in resources and advantages of by people more and more attention. These natural polymers can be used by self-assembly or crosslinking technology to form a natural polymer, thus forming the cytotoxicity of polymer, to form a stable water gel or stent with retained polymer properties (Joyce et al., 2021).

##### 2.1.2.1 Chitosan

Chitosan has excellent biodegradability and good antibacterial activity, biocompatibility, non-toxicity, and physical and chemical properties (Nakashima et al., 2006). As a result, chitosan has been widely used in the field of antibacterial treatment, such as forming antimicrobial coatings and antimicrobial scaffolds. Chitosan and its derivatives have antibacterial activity against fungi, Gram-positive bacteria, and Gram-negative bacteria, and are antibacterial materials that have attracted much attention in recent years. There are many reports showing that chitosan has excellent antibacterial activity.

**FIGURE 1:**
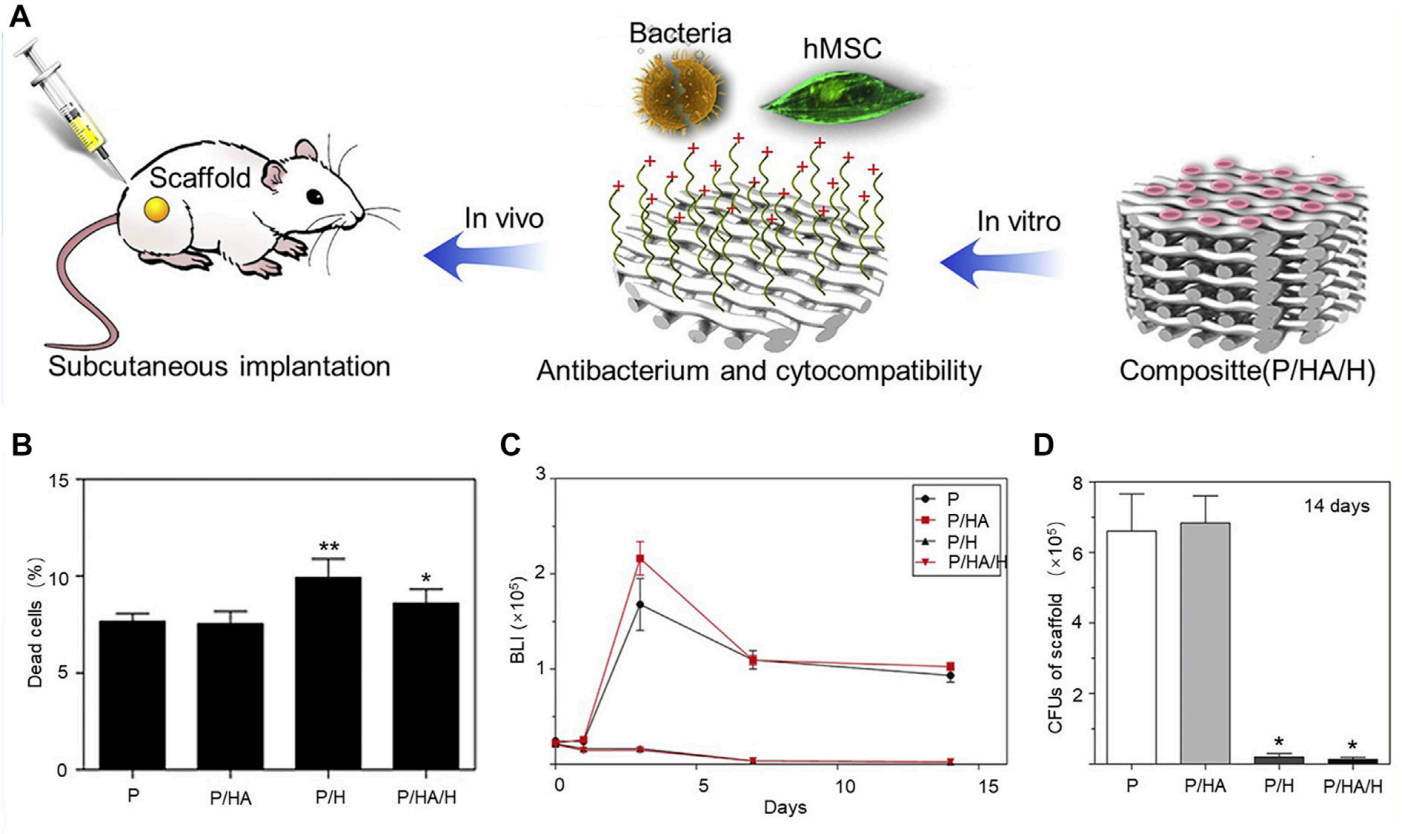
**(A)** Schematic diagram of the antimicrobial mechanism of scaffolds of HACC. **(B)** Proportion of dead cells in various scaffolds after 24-h co-culture of hMSCs on the surface of HACC scaffolds. *p < 0.01 compared to P and P/H scaffolds. **p < 0.01 compared to other scaffolds. **(C)** Mean bacterial load curve measured by *in vivo* bioluminescence (*p* < 0.01). **(D)** Quantification of bacteria in cultures from rats (*p < 0.01 compared to P and P/HA scaffolds). Copyright 2016, Elsevier Ltd. Reproduced with permission (Yang et al., 2016).

For example, quaternate chitosan, hydroxypropyl trimethyl ammonium chloride chitosan (HACC), was grafted onto a 3D-printed scaffold composed of polypropeptide coglycolactone and hydroxyapatite to design bone tissue engineering scaffolds with antibacterial and osteoconductive properties PLGA/Hydroxyapatite/HACC(P/HA/H) (Yang et al., 2016). The scaffolds grafted with HACC have good antibacterial properties and biocompatibility and, after implantation under the skin of mice (Figure 1A), the number of viable bacteria was significantly decreased in both groups of scaffolds grafted with HACC after 24 h of observation using confocal laser scanning microscopy technique. Moreover, human mesenchymal stem cells (hMSCs) were co-cultured on the surface of the HACC scaffold for 24 h, and the percentage of dead cells in the scaffold was significantly lower than in the control group (*p* < 0.05) (Figure 1B). Real-time monitoring of the bacterial photon intensity of Xen29 in the subcutaneous embedding model at days 0–14 revealed a low level of signal detected on the scaffold grafted with HACC (Figure 1C). After 14 days of scaffold inoculation, quantitative analysis of bacteria in various scaffolds by obtaining cultures in rat inoculated scaffolds revealed a significant reduction in HACC scaffold load (*p* < 0.01) (Figure 1D).

Studies also show that one step by electrophoretic deposition technique was used to prepare the antibiotics slow-release chitosan bioactive glass composite coating. In the use of vancomycin antibiotic drug loading and release of composite coating, the production of the uniform coating thickness is close to 55 µ, containing 23.7 wt% bioactive glass particles and various amount of antibiotics. Coating simulated body fluid of bioactive apatite formation showed good adhesion and cell growth (Yang et al., 2016).

Chitosan bioactive glass composite coating elution kinetics *in vitro* showed that, in the first elution steps at 1 h, approximately 40% of the drug underwent initial burst release, followed by elution for more than 4 weeks. This shows the potential of long-term drug delivery. In tests of Gram-positive *S. aureus* bacteria survival to determine the effect of vancomycin release to reduce the risk of infection, by suspending liquid containing ≥0.5 gd vancomycin on the preparation of the coating, almost no bacteria survived (Ordikhani and Simchi, 2014). This shows that the development of good biocompatibility and long-term antibiotic slow-release ability of chitosan coatings on the reduction of metal implant infection risk has a large effect.

##### 2.1.2.2 Alginate

Sodium alginate is a non-toxic, good biocompatibility, low cost natural polymer material. Alginate itself has no antibacterial properties. It is as drug carrier and tissue engineering repair material shown to have broad applications (Guler et al., 2016), such is in synthetic allogeneic heterogeneous bone grafts for human hip replacements. After being passed through the implant, the antibiotic solution is treated with a cold-dried antibiotic dressing. Alginate used in the manufacture of slow-release implantable coatings and has been characterized by spectrophotometry *in vitro* drug release curves. Study has shown that amoxicillin, ciprofloxacin, and vancomycin are combined with alginate for bone coating, which significantly prolongs the release time of antibiotics and enhances the antibacterial ability, which can support joint replacement and revision surgery (Hornyák et al., 2014). Alginate has been used in the prevention of infection for many years, but the challenge remains that not enough can be applied to human cells to form a good interaction, so alginate composites have become more popular (Zhang et al., 2016).

##### 2.1.2.3 Hyaluronic acid

Hyaluronic acid is a linear sugar glycosaminoglycan, one of the major components of the extracellular matrix, due to its good biocompatibility, hydrophilicity, antigenicity, and lubricity, and is widely used in ophthalmology, trauma, arthroplasty, plastic surgery, and other fields. Hyaluronic acid as a carrier for antibacterial materials or drugs, and shows good application potential in the field of tissue engineering (Schanté et al., 2011). Yang et al. (2022) designed and synthesized a multifunctional therapeutic material with antibacterial properties by synthesizing the porous media material ZIF-8 and applying it to load hesperidin evenly on the material surface with a layer of hyaluronic acid. This material has good stability and drug-carrying capacity and provides a slow release of antibiotics. Hyaluronic acid coated on the surface can also promote the active ingredients to infiltrate into the cells, enhancing the antibacterial ability. *In vitro* and *in vivo* antibacterial test results show a synergy between the enhanced local antibacterial activity and the material.

Although hyaluronic acid coatings increase cell membrane permeability, they also destroy the integrity of the bacteria, achieving an efficient antibacterial effect (Romanò et al., 2017). However, hyaluronic acid coatings do not appear to have significant side effects. Therefore, the application of hyaluronic acid in bone tissue engineering has good biocompatibility. Paris et al. (2019) either conjugated nisin and hyaluronic acid in a solution prior to grafting this complex onto surfaces, or grafted nisin onto surfaces previously coated with hydrolyzed hyaluronic acid, to shorten the length of the polysaccharide chains. Due to the higher local density of AMPs, when surfaces were modified with hydrolyzed hyaluronic acid they exhibited a decrease in their ability to colonize when high activation conditions were applied. The antimicrobial activity of the surface of *Streptococcus lactis* peptide was increased after immobilization and activation on hyaluronic acid. While the number of immobilized peptides is lower than the natural layer, hyaluronic acid local density is higher because of the peptide chain with a low molecular weight. Therefore, hyaluronic acid has better sterilization and prevents bacteria adhesion (Paris et al., 2017). In addition, the overall activation rate during hyaluronic acid fixation inhibits bacterial infection, so it has good antibacterial ability.

### 2.2 Inorganic materials

Inorganic materials include calcium sulfate (CS), hydroxyapatite, and metals. Among them, CS and hydroxyapatite have no antimicrobial properties *per se* and are mainly used as carriers of antimicrobial units and bone fillers in the treatment of bone infections. Metals mainly function in the form of nanoparticles.

#### 2.2.1 Calcium sulfate

CS, as a slow-release antibiotic carrier, has been widely used clinically to cure diseases such as osteomyelitis and infected bone defects. Calcium sulfate is also an important bone filling material. The implanted CS antibiotic carrier support strength promotes the repair of bone tissue and the absorbability does not cause an apparent host response in the recipient area. This creates a calcium-rich environment, whereby the calcium ions may provide some stimulus to the osteoblasts and participate in the formation of new bone (Hughes et al., 2015).

Laycock et al. (2018) used antibiotics mixed with CS for bone-gap filling beads. They modified the zone of inhibition (ZOI) test using the Kirby Bauer disc diffusion method for common orthopedic infections to investigate CS beads with different antibiotics used for 15 days. The ZOI measurement method was used to study the release changes and test the mixture’s ability to kill bacteria. The results showed that certain antibiotics combined and released from the CS to maintain an antibacterial effect. The toxin and rifampin and vancomycin bead showed antibacterial efficacy, and vancomycin with rifampicin showed drug-resistant mutation prevention. In the biofilm killing assay, all antibiotic combinations showed significant reductions in biofilm bacteria after 24 h. Exposure time is an important factor affecting bactericidal efficacy that varies by antibiotic. The CS-load ability of antibiotics can be trusted, and will not cause an explosive release of local antibiotics by heat release. This maintains the long duration of drug release and prevents soft tissue from growing into the bone defect. The biodegradation rate of CS and bone growth rate is similar, and there is no obvious inflammatory stimulation and foreign body reaction to the surrounding tissues. This makes CS very suitable for use in clinical treatment of chronic osteomyelitis (Baranes and Kurtzman, 2019).

#### 2.2.2 Hydroxyapatite

Hydroxyapatite (HA) is a new biological material; its structure is consistent with the inorganic components of bone and it occupies an important position in the treatment of bone infection, with good application prospects. Because it has good biocompatibility and bone conductibility, no cytotoxicity, no immunogenicity, and enough drug-polymer interactions, and because its degradation speed can be controlled, HA is considered to be an excellent release carrier of antibiotics and synthetic bone substitutes (Rao et al., 2016). HA can be sterilized through a variety of techniques, including gamma irradiation, gas plasma, supercritical carbon dioxide, and even steam autoclaves, and so does not cause adverse effects to structure and performance; therefore, it has significant value in clinical applications (França et al., 2013).

Many *in vitro* experiments have confirmed the therapeutic effect of HA (Stigter et al., 2004; Jiang et al., 2007; Maidaniuc et al., 2016). Additionally, nanometer hydroxyapatite (nHA) has a large specific surface area, and thus has a strong adsorption ability to carry drugs (Zhu et al., 2013). The results of chemical reduction and hot calcination methods to develop antibacterial silver nanoparticle-modified HA show strong antimicrobial activity to *S. aureus* (Bee et al., 2021). HA has satisfactory antibacterial performance; silver and gentamicin antibacterial HA-composite coatings are formed by electrodeposition and, with the addition of graphene and chitosan, improve the mechanical and adhesive properties of sedimentary composite coatings (Stevanovic et al., 2019). However, HA has the problem in that it is brittle and most products have not been used in clinical work. In general, the HA drug release system is a new and ideal functional artificial material integrating bone repair and drug treatments. The shape and particle size control of HA, the stability in the body, and the uniform distribution of antibiotics will also make HA important for research in the future.

#### 2.2.3 Metal

Since ancient times, metal has been used as an antimicrobial agent applied in medical and other fields. Metal nanoparticles are made into antimicrobial coatings or antimicrobial scaffolds for the treatment of bone infections. Copper (Cu) and silver (Ag) containers were used to store food and disinfectant, and copper salt was used to prevent fungal disease (Jiao et al., 2021). Metal, due to its excellent antibacterial effect, variety of sterilization mechanisms, and lack of drug resistance, is widely used in scientific research and clinical treatments. The most common antibacterial elements are Ag, Cu, and zinc (Zn), and others such as gallium (Ga), gold (Au), tin (Sn), and strontium (Sr) show some antibacterial properties (Miyano et al., 2007). The bactericidal mechanisms of metal nanoparticles are those of cell membrane damage, protein function inactivation, and oxidative stress. In addition to this, metals reduce bacterial adhesion. The change in surface morphology of metal nanoparticles forming antimicrobial coatings causes a change in antimicrobial activity (Bazaka et al., 2020; Zhu et al., 2021). The uneven surface of the implant increases the surface area exposed by bacterial cells and enhances the antibacterial activity. These metals provide long-term broad-spectrum antimicrobial activity as coatings (Ishikawa et al., 2017; Chouirfa et al., 2019).

Metals are mainly applied to orthopedic implants in the form of coating or alloying (Cyphert and von Recum, 2017; van Hengel et al., 2020). However, bacterial colonization of the implant surface interferes with the function of cells involved in bone formation and activates the host immune system, leading to inflammation and osteogenesis inhibition (Wei et al., 2021). Residual bacteria from a revision surgery caused by infection will further lead to implant failure. Adherence of pathogenic bacteria to the implant surface not only interferes with the function of bone-formation-associated cells, but also activates the host immune system, leading to inflammation and osteogenesis inhibition.

**FIGURE 2:**
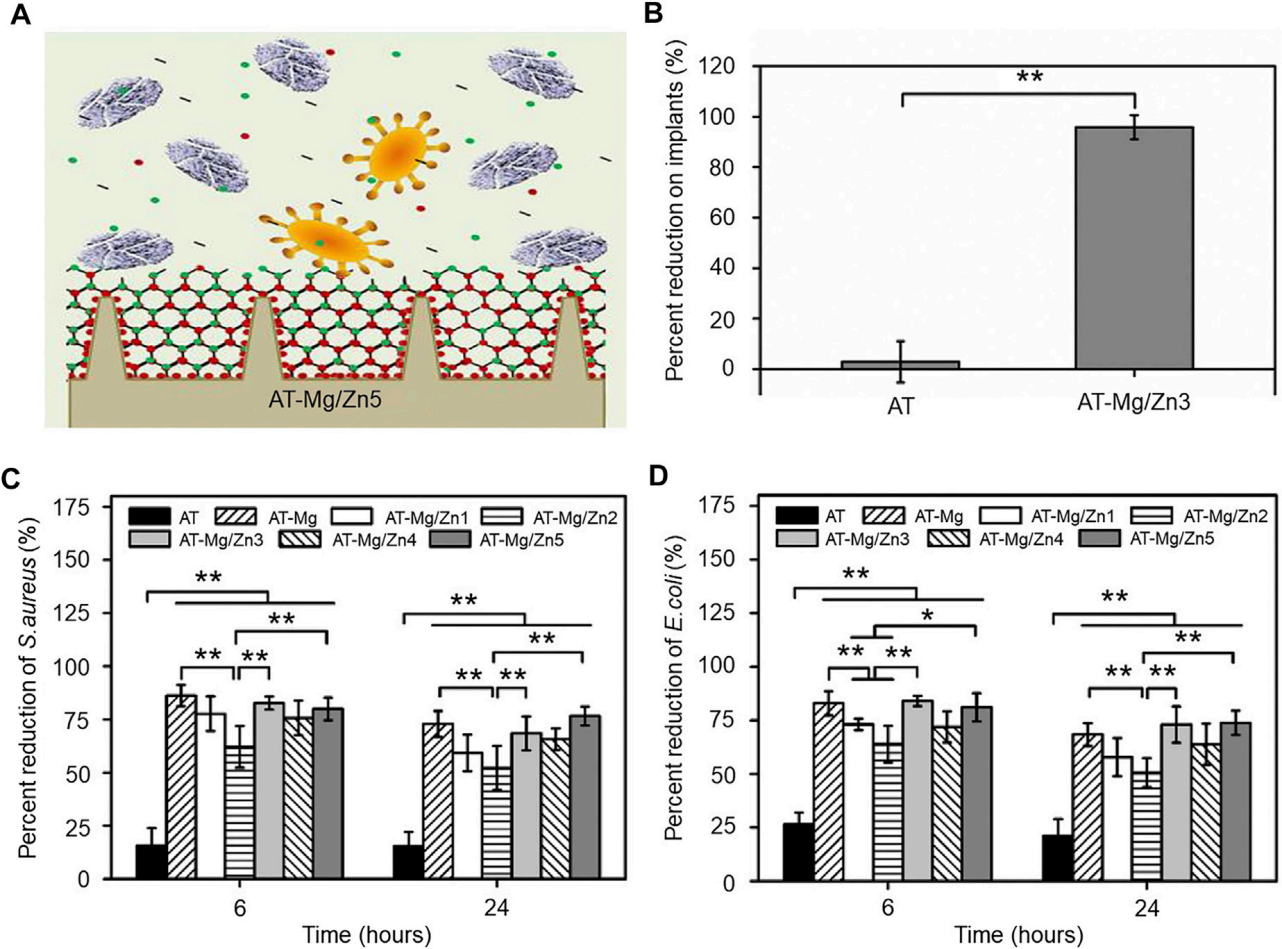
**(A)** Schematic diagram of the antimicrobial effect of the magnesium/zinc metal coating. **(B)** Quantitative statistics of implant surface bacterial inhibition on coated plate images, *n* = 8, **p < 0.01. **(C)** Antibacterial effect of *S. aureus* under different substrates, n = 6, *p < 0.05 and **p < 0.01. **(D)** Antibacterial effect of *E. coli* under different substrates, *n* = 6, *p < 0.05 and **p < 0.01. Copyright 2019, Elsevier Ltd. Reproduced with permission (Shen et al., 2019).

Shen et al. (2019) constructed a hybrid Mg/Zn metal-organic skeleton (AT-Mg/Zn) coating and investigated its antibacterial effect against *E. coli* and *S. aureus* (Figure 2A). After the coating of various materials, the surface morphology of the material shows a certain granularity and is divided into AT-Mg, AT-Mg/Zn1-5 according to the particle size. They seeded *S. aureus* on the implant surface and quantified the implant surface inhibition rate based on the coated plate images, and the AT-Mg/Zn implants showed significantly higher inhibition rates (99.9%) than the uncoated implants (over 90%) (Figure 2B). After incubation of *S. aureus* and *E. coli* bacteria on different substrates for a period, quantitative analysis by SEM images revealed a similar trend in the antibacterial effect of the antimicrobial metal coating on *S. aureus* (Figure 2C) and *E. coli* (Figure 2D).

The antibacterial properties of nanometer metal particles show great potential. Nanometer metal particles sterilization mechanisms include cell membrane damage, protein functional inactivation, and oxidative stress. In addition, the use of metal reduces bacterial adhesion. For example, the calcium-deficient calcium titanate generated by the introduction of Sr and Ag ions onto titanium (Ti) by a three-step aqueous solution treatment combined with heat, Sr, and Ag ions exhibits excellent bone growth-promoting ability and antibacterial activity (Yamaguchi et al., 2019). However, metal antibacterial materials have some problems that are impossible to ignore. These metal ions and particles enter the lymph and circulatory system and accumulate to produce cellular toxicity and carcinogenicity (Sargeant and Goswami, 2007; Teske and Detweiler, 2015).

### 2.3 Composites

Due to their limitations, single organic or inorganic materials often do not reach the requirements of clinical applications, giving rise to composite materials. Composites are formed by two or more materials and make full use of their combined advantages, good biocompatibility, strength, and antibacterial properties. The structure of the material can also affect cell function, survival, and tissue formation (Chan and Mooney, 2008). Table 1 summarizes the research progress of various antimicrobial composites in the treatment of implant-associated infections in the past decade.

**TABLE 1:** Summary of antibacterial composite materials for implant-related infections.

Compose	Antibacterial activity	Implant	Bacteria	Advantage	References
Silver ion doped ceramic nano powder	Compared to the control group, bacterial adhesion was significantly reduced	Rabbit femur	MRSA	Good biocompatibility, long-lasting antibacterial effect	Kose et al. (2016)
Gentamicin sulfate loaded chitosan with aloe (CS/AV) integrated on Ti alloy	GS drug-loaded samples showed very clear areas of inhibition for both *S. aureus* and *E. coli*	Not tested *in vivo*	*S. aureus* (ATCC 25923) and *E. coli* (ATCC 25922)	Good hydrophobicity, biocompatibility, biodegradability, and bactericidal resistance	Mohan Raj et al. (2022)
Graphitic diyne (GDY) composite TiO_2_ nanofibers	Destroying existing biofilms prevents the formation of biofilms compared to the control group	Mouse femur	MRSA	Excellent bone induction performance, good biocompatibility	Wang et al. (2020b)
D-amino acids (D-AAs) and engineered gold nanorods (AuNRs)	Significant biofilm destructive activity was exhibited compared to the control group	Not tested *in vivo*	MRSA	Cell membranes are destroyed by photothermal treatment and do not cause thermal damage to the surrounding soft tissues	Wickramasinghe et al. (2020)
Nano hydroxyapatite/collagen/CS hemihydrate (nHAC/CSH)	In the VCM/nHAC/CSH dish of the agar matrix center, the inhibition ratio of VCM/nHAC/CSH was greater than 99.8%	New Zealand rabbit femur	MRSA	Self-defining, antibacterial, porous, degradable, good mechanical properties, good osteogenesis activity	Lian et al. (2015)
CMCS and CDM-loaded mesoporous silica nanoparticles (MCM-41)	There was complete bacteriostatic activity throughout the duration of the test	Not tested *in vivo*	Streptococcus blood (ATCC 10 556™)	Enhance drug loading capacity, and maintain drug release with a fading sudden release effect, stimulate osteogenesis	Sungkhaphan et al. (2021)
Mesoporous silica nanoparticles and gelatin matrix	Compared with the control group, mesoporous silica nanoparticles and gelatin matrix composite scaffolds had a larger antibacterial inhibition zone	New Zealand rabbit radius	Gram-positive bacteria *S. aureus*	Improved bone regeneration in contaminated bone defects can be achieved	Zhou et al. (2018)
Van/PLGA is loaded into β-tricalcium phosphate scaffold	Compared with the blank stent group, CPSF has a significant antibacterial effect, and the size of the inhibition zone gradually decreases over time	New Zealand rabbit tibia	*S. aureus*	Fills the bone defect, induces osteogenesis, accelerates the reconstruction of bone tissue	Cui et al. (2020), Qiu et al. (2022), Zhu et al. (2022)
pTi/CS/HAP-Se	The composite scaffold is gradually dissolved and degraded into low molecular weight fragments after CS perfusion to exert an antibacterial effect	*In vivo* testing was not performed	*S. aureus* and *E. coli*	Osteoblasts proliferate while inhibiting tumor cell growth	Li et al. (2019)
Silk fibroins (SF) and AgNPs	Compared with pure SF stents, when the Ag concentrations were 0.1%, 0.5%, and 1%, the inhibition rate of stents increased from 51.8% to 51.8%, 72.3%, 77.5%, respectively	Rat tibia	MRSA	Promotes the growth and differentiation of various cells such as osteoblasts, bone marrow mesenchymal stem cells, and adipose stem cells, and stimulates angiogenesis	Zhang et al. (2019)
Calcium sulfate/calcium biphasic phosphate composite PMMA	This makes the local tissue pH alkaline and may interfere with bacterial growth and osteoclast activity	Rabbit tibia, rat tibia	Coagulase-positive MRSA strain	Multi-barrier, drug-eluting is biodegradable	Mistry et al. (2016), Boyle et al. (2019)
VC and GC-loaded chitosan-montmorillonite nano-clay composites (CS/MMT)	The concentration of the drug released during all time intervals was higher than the MIC value	*In vivo* testing was not performed	*S. aureus* (RSKK 1009) and *E. coli* (ATCC 25922)	There is no cytotoxic effect on fibroblasts and human osteoblast-like cells	Kimna et al. (2019)
Mesoporous silica SBA-16/HA-based	Ciprofloxacin-free SBA-16/HA and SBA-16APTES show significant antibacterial activity against *E. coli*	Swiss mice	*S. aureus*, *Pseudomonas aeruginosa*, *E. coli*, and Bacillus cereus	Good biocompatibility	Andrade et al. (2018)

To improve the combination and advantages of a variety of materials, researchers and manufacturers use coatings, nanoparticles, hydrogels, and antibacterial bracket structures to achieve a better fit. For example, nanostructured composites in the form of coatings, which embed bioactive compounds in inorganic nanostructured matrixes, incorporate biopolymers and bioactive nanoceramics that mimic organic components of the bone extracellular matrix (such as collagen) to induce bone growth (Couto et al., 2009).

**FIGURE 3:**
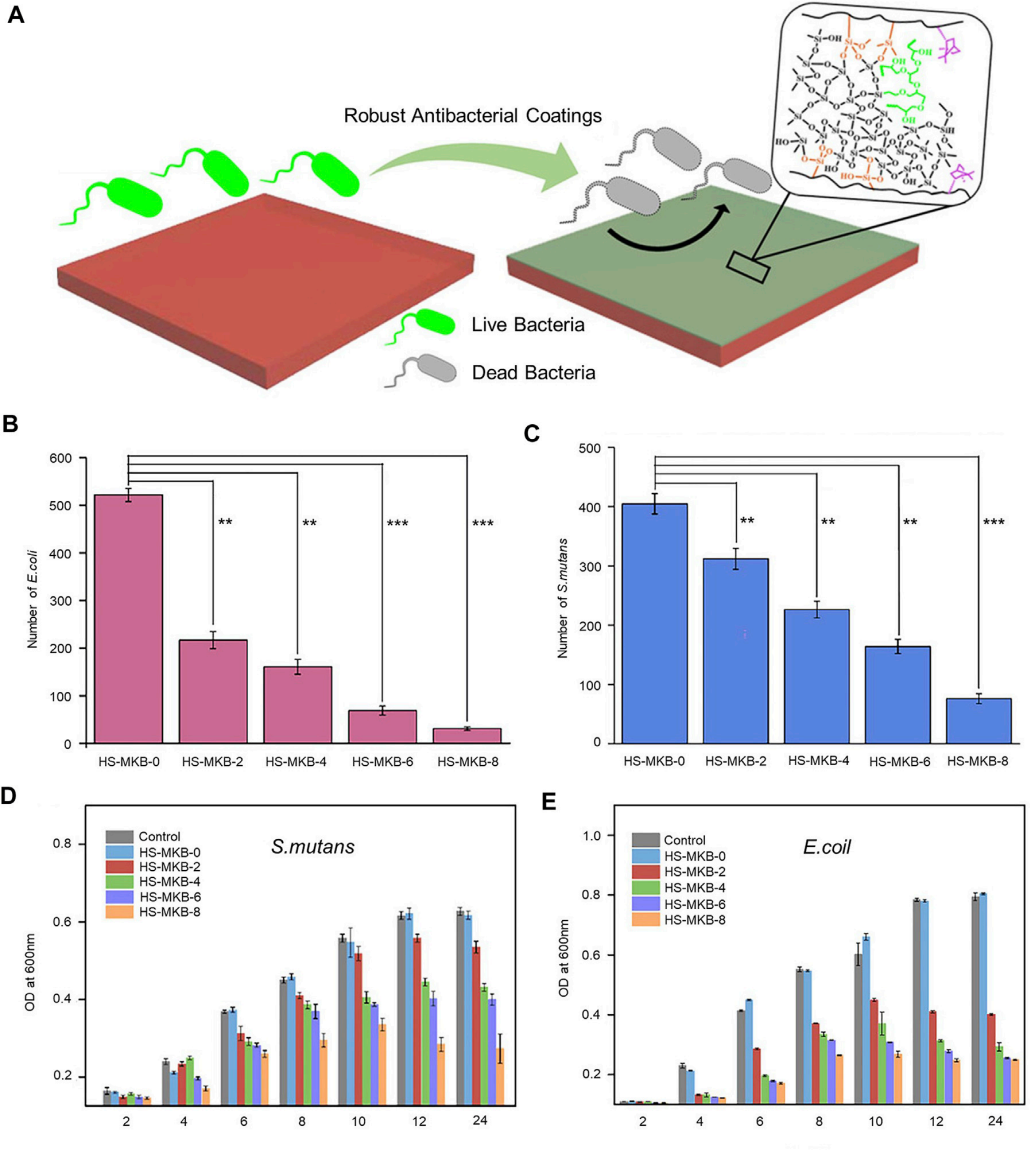
**(A)** Conceptual diagram of the organic-inorganic hybrid antimicrobial coating. **(B)** Analysis of the adhesion of *E. coln* different coatings. **p < 0.01, ***p < 0.001. **(C)** Analysis of the adhesion of *S. mutans* to different coatings. **p < 0.01, ***p < 0.001. **(D)** OD test to detect *E. coil* growth inhibition. **(E)** OD test to detect *S. mutans* growth inhibition. Copyright 2019, American Chemical Society with permission (Cheng et al., 2019).

In one study, several sets of different ratios of methyl methacrylate (MMA), 3-(trimethoxy silyl)propyl methacrylate (KH570), borneol acrylate (BA), and hybrid silica sol were mixed in a solution to prepare organic-inorganic hybrid antimicrobial coatings (Figure 3A) (Cheng et al., 2019). The relative cell growth rate of the HS-MKB-8 (MMA/KH570/BA molar ratio of 8:1:8) coating was above 85% after 24 h of incubation, as detected by the standard MTT method, indicating the low *in vitro* cytotoxicity of the coating. The bacterial inhibition ratios of the coatings were obtained by agar plates, and the HS-MKB-0 (MMA/KH570 molar ratio of 8:1) coating adhered to a large number of bacteria, while the HS-MKB-8 coating was covered with few. A high-density distribution of *E. coli* and *Streptococcus mutans (S. mutans)* was clearly observed on the surface of the slides; however, almost no bacteria were found on the surface of the HS-MKB-8. A quantitative analysis of both bacteria showed significantly less adherence to HS-MKB-0 than HS-MKB-8 (*p* < 0.001) (Figures 3B, C). The HS-MKB-BA coating was found to significantly inhibit the growth of *E. coli* and *S. mutans* by OD test (Figures 3D, E). The organic–inorganic composite coating has high light transmission, excellent mechanical properties, and good antibacterial effects on Gram-negative and Gram-positive bacteria.

Nanostructured Ag has excellent antibacterial activity, and one of the main mechanisms of bactericidal activity is cytotoxicity caused by ROS (He et al., 2012). Wang et al. (2020a) combined graphene derivatives with antibacterial metal nanostructure formations of metal-graphene composites. This increased the number of ROS generated directly, and improved oxidative stress injury caused by Ag nanoparticles. This composite also improves the dispersion of metal nanoparticles and cell contact, achieving better bactericidal activity (Rojas-Andrade et al., 2017). Luo et al. (2020) created a 3D mesoporous bioactive glass (MBG) nano fiber scaffold by single tank template assisted sol-gel doping synthesized with copper (Cu). Studies have shown that the prepared scaffold has good biocompatibility, a large specific surface area, and is endowed with the antibacterial activity of Cu, which has excellent bactericidal activity against Gram-positive bacteria. Biodegradable composite hydrogels composed of carboxymethyl chitosan and clindamycin-loaded mesoporous silica nanoparticles possess multiple antibacterial activities and induce osteogenesis by modulating alkaline phosphatase activity (Sungkhaphan et al., 2021).

By adding a hydrogel matrix nanometer conductive filler, water can be manufactured into an injectable conductive gel. These hydrogels enhance antibacterial activity and reduce cytotoxicity by enhancing electrical signal transmission and simulated physiological environment within an organism (Makvandi et al., 2020). However, this composite is limited and is likely to remain a single material. For example, bone substitutes such as ceramics and porous metals do not have enough bone induction ability (González-Sánchez et al., 2015) and there is a need to constantly adjust the proportion of compounds of various materials. Therefore, the development of composite materials has great potential.

## 3 Anti-infection and multifunctional materials

Frequent implant-related infections, leading to catastrophic results, make patients face a more complex physiological environment. With the development of composite materials, anti-infection material function has become more diversified. They have specific bioactive components as a way to provide specific functions (such as Biocompatible, promotes angiogenesis, osteogenesis, promotes coagulation and clears dead bacteria). Here, we discuss the different functional antimicrobial materials and their associated bioactive components separately. Some of the biomaterials with special functions are listed in Table 2.

**TABLE 2:** Functions and applications of bioactive components in biomaterials.

Bioactive ingredients	Function	Basic antibacterial materials	Applications	RIS
Serine protein (SF)	Excellent mechanical properties, good biocompatibility and biodegradability	Gentamicin, AgNPs	Vancomycin-loaded SF-AgNPs scaffolds	Zhang et al. (2019)
Zn^2+^	Promotes angiogenesis and osteogenesis	Zeolite imidazole acid skeleton-8 (ZIF-8)	Preparation of ZIF-8 nano-organic skeleton by solvent method	Zhang et al. (2021)
PLGA	Biodegradable, with good biocompatibility	AgNPs, Stainless steel alloy	Implants with PLGA/AgNPs coating	Liu et al. (2012)
Poly(3-hydroxybutyrate-co-3-hydroxypentanoate) (PHBV)	Excellent biocompatibility and biodegradability	CuO, AgNPs	PLA- and PHBV composites containing nanoparticles	Mazur et al. (2020)
Genipin(GP)	Good compatibility and low cytotoxicity	Multifunctional carbon dots (CDs)	A novel nitrogen co-doped carbon dot-dye wood covalent conjugate (N-CDs-GP) was synthesized by hydrothermal method	Chu et al. (2020)
Vascular endothelial growth factor (VEGF)	Vascular endothelial cell migration, proliferation	Cu^2+^	3D printed hybrid scaffold with bionic hierarchical porous structure	Wu et al. (2020), Zhang et al. (2022)
Cu^2+^	Stimulates angiogenesis and collagen deposition	Cu^2+^	Biocomposites containing copper Mesoporous bioactive glass	Wang et al. (2016)
Bioactive glass (BG), Ga	Stimulates expression of osteoblast genes and stimulates angiogenesis, Inhibit bone resorption and increase calcium deposition in bone tissue	Ga, Carboxymethyl cellulose (CMC), dextran	Ga-containing BG particles form hydrogel composites	Keenan et al. (2017)
Mg^2+^	Local Mg-rich environment improves osteoblast adhesion rate, promotes new bone production and regulates osteoblast signaling	Mg^2+^, TiO_2_	Preparation of magnesium-doped titanium dioxide microporous coatings on the surface of Ti implants	Zhao et al. (2019)
Graphdiyne (GDY)	Good osteogenic ability and promotes osteoblast adhesion	TiO_2_, GDY	Assembly of GDY onto TiO_2_ as a coating for titanium implants by changing the surface charge of GDY and TiO_2_	Wang et al. (2020a)
Mesoporous silica spheres	Absorbs water from wounds, promotes clotting factor activation, and is degradable	Ca^2+^, Ag^2+^	Calcium-silver doped ordered mesoporous silica spheres for antibacterial and hemostatic applications	Dai et al. (2009)
Chitosan	Positively charged chitosan tends to adsorb negative proteins, which leads to platelet activation and thrombosis	Ag-NPs	Preparation of chitosan nanofibers by electrostatic spinning	Dong et al. (2018), Cheng et al. (2020)
Carboxymethyl kappa-carrageenan	Interacts with blood proteins to promote clotting and absorbs exudate from wounds	Carboxymethyl kappa-carrageenan	Mixing Carboxymethyl kappa-carrageenan with poly(vinyl alcohol) to introduce antibacterial activity and procoagulant activity into nanofibers	Madruga et al. (2021)
Poly(N-isopropylacrylamide)	Unique temperature responsiveness with improved wettability and adhesion properties	Lysozyme	Modulation of spatial hiding and exposure of adsorbed lysozyme by temperature-triggered hydration and conformational changes	Yu et al. (2014a)
Poly(methacrylic acid)	Imparts pH responsive	Lysozyme	The poly(methacrylic acid) chain imparts pH-responsive properties to the system for stepwise modulation of surface and lysozyme/bacterial interactions	Wei et al. (2016)
Poly(carboxybetaine methacrylate)	An amphoteric polymer that strongly interacts with water to release of dead bacteria	Ag-NPs	Silver nanoparticles embedded in a polymer matrix capable of killing bacteria on contact and releasing dead bacteria under moist conditions	Hu et al. (2013)

### 3.1 Antibacterial materials with good biocompatibility

Biocompatible materials are defined as those that perform their desired functions in an organism without risking tissue damage, cytotoxicity, or rejection by the immune system, and without causing inappropriate local or systemic effects or reducing the ability of the host to respond and interact with living systems (Williams, 2008; Williams, 2017). Orthopedic biocompatibility and antibacterial materials now reduce the interference of bone and surrounding tissue homeostasis to reduce the release of any harmful products for the bone and surrounding tissue; optimize the biomechanics of bone deposition speed, quality, and environment; and improve the speed of bone adaptation and efficiency. Zhang et al. (2021), using a solvent and diethanolamine template method, prepared a corn lithium base with a typical structure of zeolitic imidazolate framework (ZIF)-8 antibacterial nanomaterials. The material has a larger specific surface area and smaller aperture, good biocompatibility and antibacterial properties, and significantly improves the antibacterial activity of *E. coli* and *S. aureus*. Antibacterial metal nanoparticles added to other materials is also a hotspot of current research, and antibacterial nano metal concentration significantly affects the surrounding tissue.

Nanoparticles, due to their small size and unique properties, are considered to have a high degree of mobility, a variety of proteins, and a strong nucleic acid structure formation in the biological environment, which may enhance or limit several cell functions (Liu et al., 2008). For example, (Liu et al., 2012) found that Ag nanoparticles added to a coating promoted osteoblast proliferation and maturation. Conversely, Zhao et al. (2011) and De Giglio et al. (2013) found that adding a coating of Ag nanoparticles increased the cytotoxicity of osteoblasts. A study using mercaptododecylphosphonic acid (MDPA) found that, after the occurrence of the AgNO_3_ reaction, a MDPA/AgNO_3_ nano coating forms, which prevents bacterial adhesion and biofilm formation on medical planted instruments without affecting biological compatibility (Tîlmaciu et al., 2015). Others studies used polylactic acid (PLA) and poly 3-hydroxybutyrate-co-3-hydroxyvalerate (PHBV) in the formation of a composite material by adding silver (Ag), and found that it does not significantly affect the mechanical properties and biodegradation rate, while increasing the protective barrier against bacteria, with excellent biocompatibility (Mazur et al., 2020).

Genipin (GP) is a biological crosslinking agent extracted from the fruit of *Gardenia jasminoides* that readily forms stable cross-networks and can provide good biocompatibility and low cytotoxicity. A novel nitrogen co-doped carbon dots (CDs)-genipin covalent coupling (N-CDs-GP) compound was synthesized by the hydrothermal method to couple quantum carbon nanomaterial CDs with GP to combine the features of excellent water solubility, high photostability, and low cytotoxicity (Chu et al., 2020). To verify the biocompatibility of N-CDs-GP, cells excited with CLSM488 and 543 nm laser pulses to an N-CD-GP aqueous solution exhibited bright green and red fluorescence, and the emission of multicolor fluorescence without any identifiable morphological defects, indicating that N-CDs-GP has reliable biocompatibility. Additionally, to verify its hemolytic activity, erythrocytes in phosphate-buffered saline were used as a negative control and erythrocytes in distilled water were used as a positive control. Compared with the negative control, erythrocytes in solution with a high concentration of N-CD exhibited a roughly normal biconcave shape, and erythrocytes in solution with N-CDs-GP exhibited a typical biconcave shape. The antibacterial effect was further demonstrated by the bacterial morphology observed by scanning electron microscopy. By comparing the *S. aureus* count and healing at the infection site in the mouse model, it was found that the infected site healed well after treatment and the amount of *S. aureus* in the infected tissue was much lower than that in the control group. A good combination of antimicrobial properties and biocompatibility of the material was obtained. Compared with traditional materials, this composite coating has good biocompatibility and antibacterial activity, and the superior features may be found in the development of potential orthopedic implants and antibacterial devices.

### 3.2 Antibacterial materials with pro-angiogenic effect

The fracture healing process is affected by the blood supply of the fracture, stability, and the healing site. The influence of nourishing blood vessels, once damaged, will seriously affect the healing of the fracture, and even cause infection (Claes et al., 2012). Although traditional orthopedic implants show effective bactericidal activity and are composed of antibacterial materials, they lack the ability to promote angiogenesis. Several attempts have been made to develop and promote angiogenesis of antibacterial materials to solve this problem. Recently, a study on the immobilization of vascular endothelial growth factor (VEGF) proved than repeated doses of soluble VEGF play a role in angiogenesis (Leslie-Barbick et al., 2009; Zhang et al., 2022). VEGF, used in the development of advanced bioactive scaffolds, not only promotes new bone formation and growth, but also induces angiogenesis (Kim et al., 2012; Wu et al., 2020).

Wu et al. (2013) prepared a load of ibuprofen copper-MBG (Cu-MBG) stents which, together with ion extract, stimulate human bone marrow stromal cells (hBMSCs) hypoxia inducing factor (HIF)-1 and the expression of VEGF to enhance angiogenesis and, through the relevant gene expression, improve bone stimulation of osteoblast. The sustained drug release load, and the presence of Cu_2+_, ensured good antibacterial properties (Borkow et al., 2008). Other studies have shown that cellulose nano fibrosis (NFC) and the formation of Cu-MBG composite aerogel materials enhance angiogenesis (Wang et al., 2016). A biomass chitosan hydrogel micro needle array (CSMNA) was recently developed with VEGF by temperature sensitive hydrogel coating in CSMNA pores. The intelligent release of drugs can be controlled by the temperature increase caused by the local inflammatory response, which can promote inflammation inhibition, collagen deposition, angiogenesis, and tissue regeneration in the process (Chi et al., 2020).

In original titanium implants, vulnerable to bacterial infection, the effective method to reduce implant failure is to modify the surface of the implant, making it suitable for bone forming cells with simultaneous anti-infective properties. Titanium is combined with the substrate surface by covalent grafting of dopamine, carboxymethyl chitosan (CMCS) or hyaluronic acid-catechins, and VEGF coupling for functional polysaccharide transplantation. CMCS and functional titanium promotes angiogenesis, strengthens the function of osteoblasts, and simultaneously reduces bacterial adhesion (Hu et al., 2010). Similarly, synthesis polymers can be easily coated on polydopamine modification of the titanium surface, and loaded with antibiotics for slow release (Zeng et al., 2018). These features of implantable medical devices provide a new function to prevent infections by the promotion of angiogenesis.

### 3.3 Antimicrobial material with osteogenic effect

With the increase of orthopedic implant material use, we face the serious situation of bone infection treatment, and urgently need to improve the osteogenic and antimicrobial capabilities of implantable antimicrobial materials in the application of orthopedic implants, as biological complications such as insufficient bone mass and postoperative infection lead to treatment failure. The implantable material is in direct contact with the bone interface, and the promotion of osteogenesis is key for the antibacterial material to exert its physiological function. However, traditional implantable materials do not have this capability. Emerging antibacterial osteogenesis multifunctional materials are designed to compensate for these shortcomings, in both the osteogenesis phase and in the long-term support of the local structure.

Gallium doped materials enhance the osteogenesis effect in biologically active substances and improve antibacterial activity (Verron et al., 2012; Sanchez-Salcedo et al., 2018). Gallium added to biomaterials holds great potential to treat bone-related diseases, because it can be effectively transferred to the required area at a controlled rate. In addition, it can be used as a potential alternative to antibiotics, as well as in the initial and late stages of wound healing to inhibit infection (Keenan et al., 2017; Crunkhorn, 2018).

Chen et al. (2022) used alkali heat treatment of titanium on the implant surface layered with an Mg-Ga double oxide nano coating. The coating significantly promoted the autophagy activity of mesenchymal stem cells, induced osteogenetic differentiation, and suppressed the generation of osteoclasts. In addition, the researchers found that the zinc mixed with ZnO_2_ delicately balanced the in antibacterial function and osteogenesis (Cheng et al., 2015). The titanium metal implant surface formed a nanostructure coating that stimulated macrophage elongation, promoted the production of proinflammatory cytokines, and induced macrophages to anti-inflammatory M2 phenotypes. This promote osteogenesis differentiation, such as cell adhesion, alkaline phosphatase activity, mineralization of the extracellular matrix, and raised the osteogenesis related gene expression (Chen et al., 2021; Jiang et al., 2022). Zhao et al. (2019) showed that the plasma electrolytic oxidation porous Mg-TiO_2_ covered Ti surface not only promote osteoblast adhesion, proliferation, and differentiation, but also inhibited Staphylococcus engraftment and growth.

**FIGURE 4:**
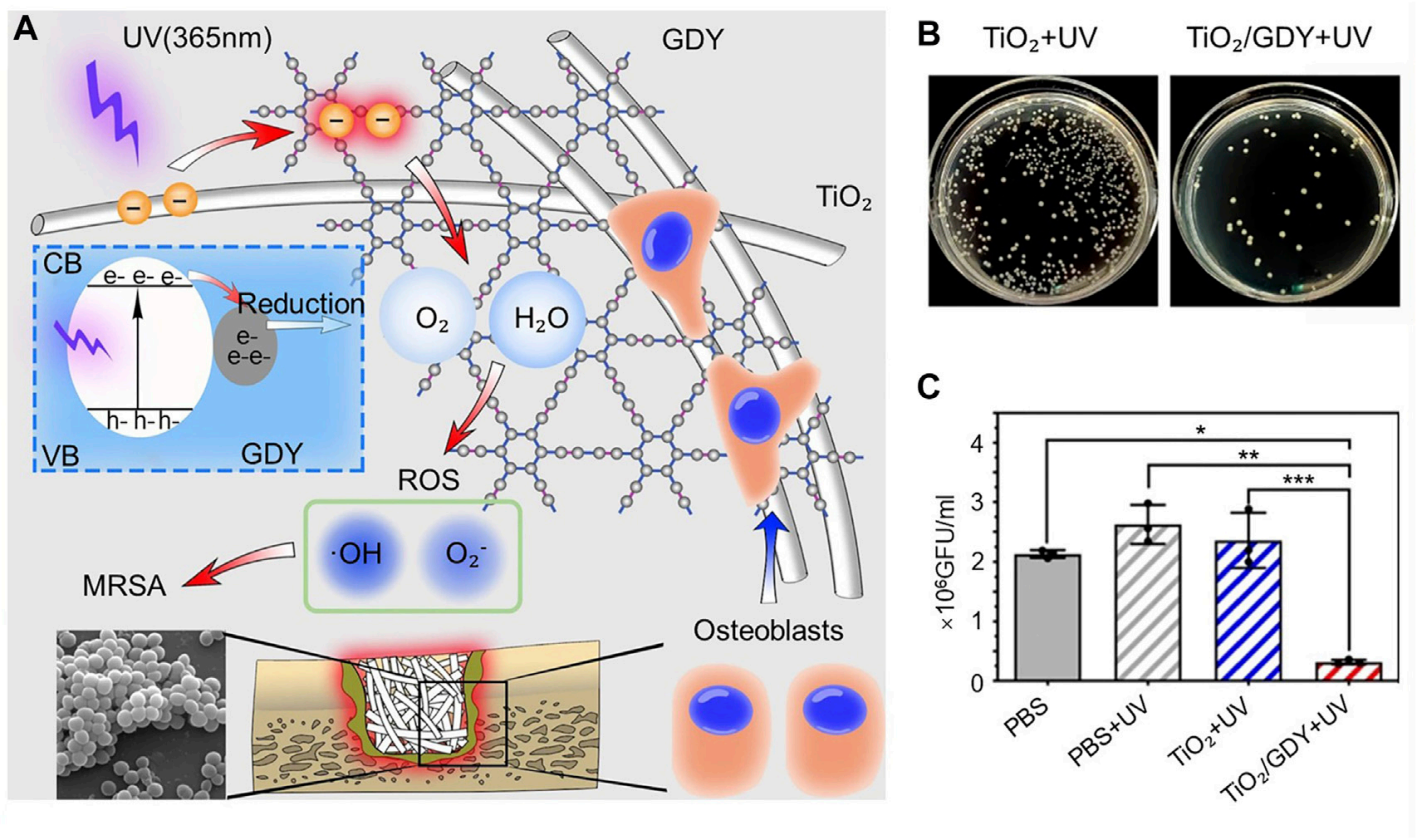
**(A)** Schematic representation of the function of TiO₂/GDY and its application in orthopedic implant infections. **(B)** Bacterial colony plate counts in implant femoral grinds of the infection model. **(C)** Quantitative analysis of bacterial colonies in the infection model. *p < 0.0002; **p < 0.0001; ***p < 0.0001). *Copyright © 2020, The Author(s).* Reproduced with permission (Wang et al., 2020b).

The hydrophilic nature of graphene can lead to enhanced expression of osteogenic genes. One study assembled titanium alloy nanomaterials by using graphitic diyne (GDY) and titanium combined to form titanium alloy nanomaterials. GDY enhances the osteogenic effect by promoting osteoblast adhesion to the nanomaterials, and UV irradiation (365 nm) leads to more ROS production for sterilization (Figure 4A) (Wang et al., 2020b). Bacterial colony plate counts were performed to detect colonies in an implanted femoral grinding solution of an infected model. It was found that the TiO₂/GDY + UV group had significantly less MRSA than the TiO₂ + UV group (Figures 4B, C). By SEM scanning of the implanted nanomaterials, the cells appeared to adhere firmly to the surface of the GDY-modified material. After observing the pathological tissue of femoral bone infection using HE staining, the investigators found that the TiO₂+UV group had obviously infected and necrotic areas in the bone defect area (orange dashed line), while the TiO₂/GDY + UV group had little inflammatory cell infiltration in the bone defect area. Multifunctional bone-promoting antibacterial materials have been intensively studied, and still have great potential.

### 3.4 Antimicrobial material with coagulation effect

Promotion of the blood-coagulation function of antibacterial material for trauma care is very important, as it prevents acute bleeding to save lives, and prevents bacterial proliferation in the early stage of open or acute tissue injury (Alam et al., 2012; Yang et al., 2021). To promote organism coagulation (Wang et al., 2019b), antibacterial hemostatic materials absorb moisture, enrich the blood components, initiate the endogenous or exogenous coagulation system, and induce rapid solidification, eventually forming a blood clot to limit bleeding (Yan et al., 2017; Krishnadoss et al., 2019).

Mesoporous silica is an emerging hemostatic material that absorbs water, promotes clotting factor activation, and assists in biodegradation (Dai et al., 2009). According to a study (Shen et al., 2020), cationic groups function to make use of the antibacterial properties of the polymer, with mesoporous silica coordination of superabsorbent hydrogel formation showing good potential utilization value. Cationic polymers absorb the negatively charged red blood cells, blood fibrinogen, and plasma protein, which constitute the hemostatic properties of cationic polymers (Qin et al., 2020). Structure modifications do not usually change the basic properties of the cationic polymer, but introduce a new function. For example, modified cationic chitosan has improved antibacterial ability and hemostatic properties (Sahariah and Másson, 2017). Lu et al. (2017) found that incorporation of Ag/ZnO further enhances the capacity of cationic chitosan blood coagulation, because Ag denatures the anticoagulant protein, thus affecting the organism coagulation pathway (Kapadia, 2005). The hemostatic activity of medical nanoscale antibacterial fibers has garnered wide attention because the characteristics and chemical properties of these fibers promote blood clotting (Fischer et al., 2008).

Blood clot formation is composed of platelet activation and aggregation and a multi-step coagulation cascade, which leads to the aggregation of fibrinogen and the formation of a crosslinked fibrin fiber network. The nano fiber structure of can be affected by rapid dehydration, as blood acts as a molecular sieve by producing a mechanical barrier, leading to blood clots. This process accelerates the coagulation cascade and promotes hemostasis (Giri Dev and Hemamalini, 2018). The excellent antiplatelet adhesion ability of polymers in the formation of nanofibers will trigger increased platelet adhesion and activation (Nakielski and Pierini, 2019). For example, when using chitosan nano powder (NCTS) in PLA, with the help of electrostatic spinning into nanofibers, the NCTS/PLA activated partial thrombin time is longer than in the PLA; more NCTS lead to a longer clotting time (Dong et al., 2018). In addition (Cheng et al., 2020), used electrostatic spinning to identify uniform distribution of silver nanoparticles (Ag-NPs) in PLA and gelatin (Gel) composite fibers forming inside the PLA/Gel/Ag composite. This showed the promotion between the proliferation and induction of osteogenesis of the mesenchymal stem cells, with good bone conductibility and hemostatic properties. Traditional hemostatic strategy depends on the use of blood components, such as fibrinogen and thrombin. These components come with a high cost and short shelf life. To overcome these limitations, the potent protease, thrombin, should be avoided. Qin et al. (2015b) photoconjugated thrombin-receptor-agonist-peptide-6 (TRAP6) onto cyto-compatible polyvinyl alcohol hydrogels *via* efficient thiol-norbornene photoconjugation, which preserve the platelet-activating activity, for safe hemostasis and infection prevention. Introducing antibacterial and procoagulant activities into nanofibers is a focus of current research regarding procoagulant antibacterial materials.

Recently, studies have used carboxymethyl-copper-carageen glue mixed with poly (vinyl alcohol) nanofibers (PVA-CMKC), to increase blood clotting and antibacterial activity, and promote platelet adhesion and activation (Madruga et al., 2021). Because the nanofibers of CMKC have rigid cell walls, neither Gram-positive nor Gram-negative bacteria readily adapt to the nanoscale features, which may kill bacteria on the nanostructured surface (Madruga et al., 2020).

### 3.5 Antimicrobial materials with intelligent “kill and release” effects

Traditional antibacterial strategy is divided into two kinds: one is to kill adhering bacteria through the material’s natural bactericidal properties or fungicides (Xue et al., 2015; Pinho and Piedade, 2020); and the other bacteria-repellent material works through its properties that prevent bacterial adherence (Song et al., 2015; Moradi et al., 2016; Ramachandran et al., 2019). However, both types have limitations. The bactericidal properties or fungicide kill the bacteria, but the debris usually pile up at the local site, causing a lowering in germicidal efficacy. It is possible that this provide nutrients for subsequent bacterial adhesion and causes an immune response. In contrast, flooding the bacterial material without sterilization causes adhesion of the bacteria to the surface, which grows into a biofilm. Therefore, combining the functions of repellent and sterilization on one surface is the focus of current research. Bacterial killing and the release of specific functions may interfere with each other, and usually requires intelligent switching in function. These smart antibacterial surfaces kill the bacteria attached to the surface, and then switch to control the temperature, pH, humidity, or other external factors that stimulate the release of dead bacteria and its debris. This maintains kill efficiency (Wei et al., 2017).

#### 3.5.1 Intelligent control components: Temperature

A common temperature control switch is poly (N-isopropyl acrylamide) (PNIPAAm), a heat responsive polymer. PNIPAAm modified antibacterial material surfaces have a unique temperature control surface with improved wettability and adhesion performance (Frazar et al., 2020; Nagase, 2021). Because the surface of the lysozyme has been shown to effectively destroy bacteria cell membranes, its effectiveness is limited by dead bacteria and debris accumulation (Düring et al., 1999). Yu et al. (2014a) prepared PNIPAAm nanopatterned brushes by interference lithography, and lysozyme was combined with PNIPAAm to adsorb to the polymer-free regions of the substrate between the brushes. The sterilization and drainage capacity of the surface of the hybrid was switched according to temperature. Yu et al. (2014b) also used resonant infrared matrix-assisted pulsed laser evaporation (RIR-MAPLE) based on quaternary ammonium salts and sterilization of PNIPAAm thin film deposition as an antibacterial film on the material. Research shows that, when the temperature is higher than the lower critical solution temperature (LCST), the multifunctional film killed a large number of adherent bacteria. The dissolution of PNIPAAm components, when the temperature is lower than the LCST flush surface, leads to the expansion of the PNIPAAm brush to release the dead bacteria, thus forming a temperature control switch. The materials are then reused.

#### 3.5.2 Intelligent control element: pH

**FIGURE 5:**
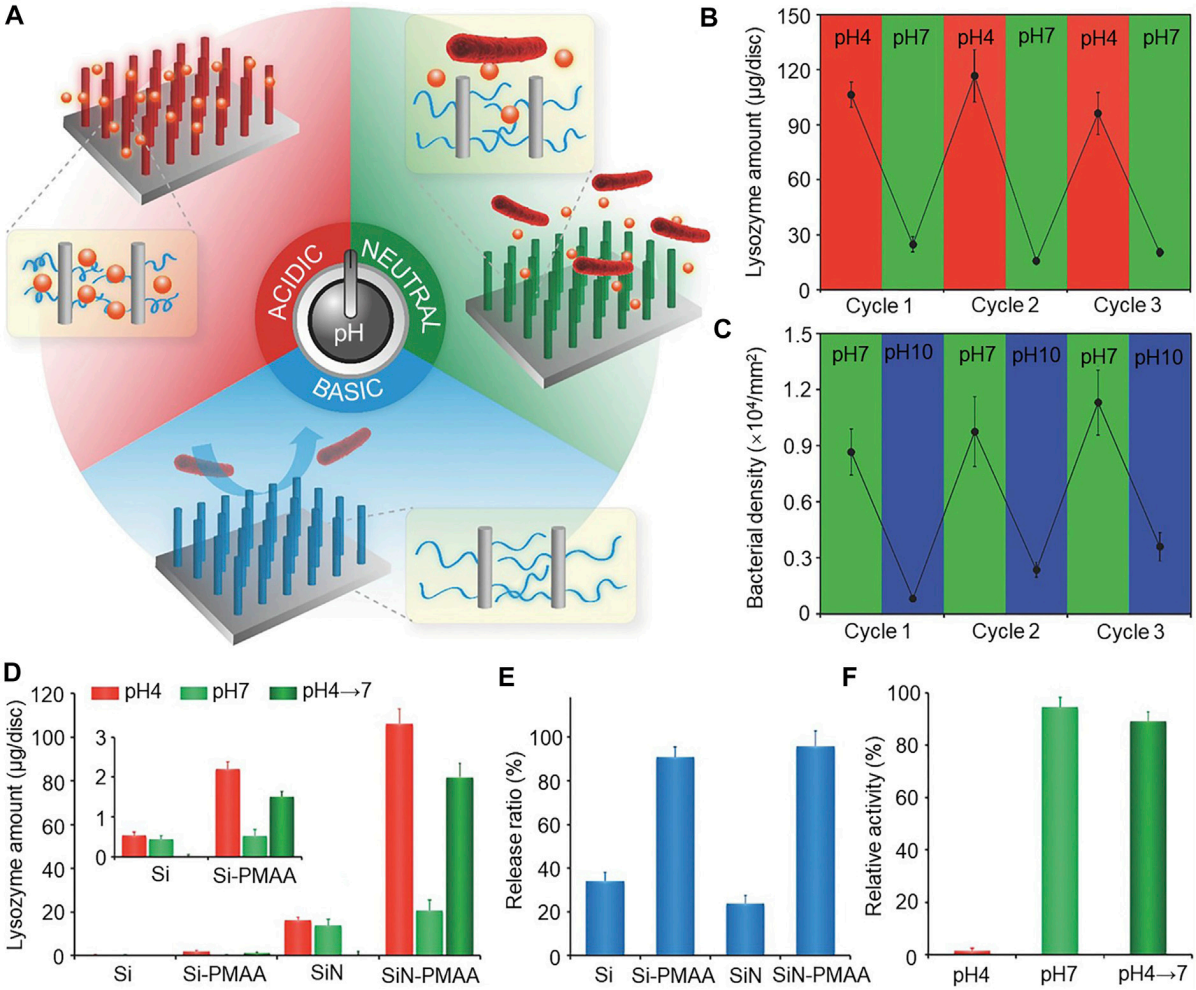
**(A)** Schematic diagram of the smart antimicrobial surface, which can function according to pH change. **(B)** Reversible binding of lysozyme by pH switching of the SiN-PMAA surface. **(C)** Reversible attachment of *E. coli* by pH switching on the SiN-PMAA surface. **(D)** Release of lysozyme in solution when the pH changes from 4 to 7. The inset shows the Si and Si-PMAA surfaces. **(E)** Comparison of lysozyme release ratios from pH 4 to 7 on antimicrobial surfaces. **(F)** Comparison of the relative enzymatic activity of free lysozyme at pH 4 and 7. Error bars represent the standard deviation of the mean (*n* = 3). *Copyright © 2016, The Author(s).* Reproduced with permission (Wei et al., 2016).

pH response polymer poly(methyl acrylic acid) (PMAA) is a weak electrolyte, wherein the charge density and conformation depend on pH. When it is immobilized on the material surface, changes in ambient pH leads to changes in wettability and surface charge, which in turn lead to changes in bio-adhesion to achieve regulation (Negim et al., 2014; Kumar et al., 2021). The preparation of SiN-PMAA surfaces with PMAA vertical silicon nanowire arrays (SiN) show that, in an acidic environment, the material can bind large amounts of lysozyme. In a neutral environment, the surface of the material releases the loaded lysozyme to kill the attached bacteria in the vicinity. In an alkaline environment, the material releases the attached dead bacteria to keep the surface clean for subsequent loading of new lysozyme, allowing the material to be reused (Figure 5A). The reversible attachment of lysozyme and *E. coli* was achieved on the SiN-PMAA surface by pH switching (Figures 5B, C). By radiolabeling method, it was found that although Si-PMAA in the control group showed a similar protein release rate as SiN-PMAA (Figure 5F). However, the lysozyme released in solution when the pH changed from 4 to 7 was found to have an extremely high binding capacity at a lysozyme pH of 4 (Figure 5D). The relative enzyme activity of free lysozyme at pH 4 and pH 7 differed significantly (Figure 5E) (Wei et al., 2016). The process does not affect the lysozyme activity and can, therefore, undergo repeated use.

There has been a study using a layered structure of polymer brushes to fabricate pH-controlled PMAA and antimicrobial peptide (AMP) dual antimicrobial surfaces (Moradi et al., 2016). The PMAA in the outer layer uses an intelligent control element, and the AMP uses the fixed inner layer original for sterilization. When bacteria colonize the surface bacteria, it triggers acidification of the outermost PMAA chain, which collapses, exposing potential sterilization AMP and killing the bacteria. In addition, when the environmental pH value increase in the PMAA, the hydrophilic chain is restored, and the dead bacteria are released. This process can be repeated with no additional reloading of the antimicrobial agent.

#### 3.5.3 Intelligent control components: Zwitterion polymer

Zwitterionic polymers refer to those with an equimolar number of anionic and cationic groups evenly distributed along the chain. Zwitterionic compounds with mixed anionic and cationic end groups have strong antifouling properties and are often reported for modification of various surfaces to enhance the hydrophilic, antifouling, and antibacterial properties (Li et al., 2020). Grafted zwitterionic polymer surfaces, such as betaine and choline phosphate, strongly bind to water molecules through electrostatic interaction in aqueous solutions, to form a stable hydrated layer, and show excellent drainage performance (Mary et al., 2007; Sin et al., 2014). The hydration behavior of sulfobetaine brush array structures have features that make it have excellent drainage capacity (Xiang et al., 2018).

In an aqueous environment, zwitterionic polymers form a hydration layer for significant inhibition of planktonic bacteria adhesion, accumulation of dead bacteria, and drainage. In dry conditions, when the zwitterionic layer collapses, the polycationic layer exhibits bactericidal properties against adherent bacteria (Yan et al., 2016). For example, tyramine conjugate sulfobetaine synthesis polymers react with tyrosinase grafted to the surface of polyurethane, and impart antifouling, antibacterial, and lubricating properties to the modified surface through water contact (Kwon et al., 2017; Li et al., 2020). nAg synthesized poly(sulfobetaine methylacrylate) (pSBMA) and poly(carboxybetaine methylacrylate) (pCBMA) zwitterionic polymers scatter good silver nanoparticles embedded in the pCBMA matrix. pCBMA-CB-Ag kills the bacteria by contact and releases the dead bacteria in wet or humid conditions (Hu et al., 2013). This surface has a controllable switch based on humidity sterilization, drives the mechanism to kill bacteria on the surface when dry, and the removal of dead bacteria in wet environments. This makes these polymers effective in anti-infection implants and medical equipment.

## 4 Conclusion and future perspectives

The global orthopedic market is still expanding, and implant placement is a common treatment method therein. Once orthopedic implants become a hiding place for bacteria and infections, bone destruction and bacterial infiltration will occur, and bone regeneration will be insufficient, which will inevitably lead to insufficient local support and the formation of sequestered bones. Despite progress in this field, the development of implantable antimicrobial materials capable of preventing bacterial proliferation is a research challenge that must be overcome due to the decline in antibiotic effectiveness, global superbugs, and antibiotic-resistant bacteria. Therefore, the implant itself requires lasting antibacterial ability and versatility.

We reviewed the latest progress in the field of biomedical antibacterial materials. Firstly, we introduced the respectively organic and inorganic antibacterial and composite materials being used. Antimicrobial materials include the direct and indirect materials against infection. These materials show bactericidal or bacteriostatic activity through slow release of antibacterial agents. However, these materials have some limitations such as: 1) the use of PMMA non-biodegradable material carries that risk of bacterial biofilm; 2) metal ions and particles enter the lymphatic and blood circulation systems, and accumulate in cells and tissues, resulting in cytotoxicity, genotoxicity, and even carcinogenicity; 3) HA and calcium phosphate may be brittle, and not suitable for bearing orthopedic applications; 4) polymer antibacterial activity duration of this biological material is limited, and part of the material depends on antibiotics; and 4) composite materials may reduce cytotoxicity by reducing the concentration of nanoparticles of a single composition, but may still have the limitations of a single material.

Despite the above limitations, the available data for implanting antibacterial material is encouraging. We also outlined currently available antibiotic delivery devices in the application of composite materials. A combination of approaches could be an excellent way to increase the effectiveness of novel antimicrobial materials, and delivery of antibiotics through novel structures and devices could be an excellent option to overcome some practical limitations of commonly used coatings, such as their specificity. We look forward to future research defining an orthopedic antimicrobial biomaterial as that designed to work with permanent orthopedic implants to provide local antimicrobial treatment and maintain implant function.

We also reviewed the most advanced multifunctional antibacterial materials and surfaces. These have biocompatibility, are angiogenesis, osteogenesis, and blood-coagulation promoting, and have “smart killing” capabilities. Biocompatibility materials that properly perform the required function do not result in tissue damage, cell toxicity, risk of rejection, as well as local or systemic effects. They also do not cause undue appropriate host response in the execution of their functions. This is key of the related materials research that cannot be ignored.

These materials must promote angiogenesis of antibacterial material, promote osteogenesis, and help the body create a suitable environment for organization. The process of cell proliferation is essential to promote fracture healing and tissue repair at the surgical site, and antibiotics are often used in implant materials to prevent implant infections. However, excessive use of antibiotics significantly reduces the function of osteoblasts and bone marrow mesenchymal stem cell vitality and proliferation, which will damage the healing process (Philp et al., 2017; Braun et al., 2020). Therefore, the development of novel antibacterial materials that promote osteogenesis has good research prospects.

Antibacterial materials that promote blood-coagulation function to promote coagulation of organisms, activate endogenous or extrinsic coagulation systems, induce rapid hemostasis, and prevent bacterial proliferation in the early stage of acute tissue injury or open fractures. Bacterial killing and release of specific functions may interfere with each other, usually requiring intelligent switches of function. Intelligent antibacterial surfaces kill the bacteria attached to the surface, and through control of temperature, pH, humidity, or other external factors, such as the stimulating release of dead bacteria and debris, maintain kill efficiency. These processes are efficient and repeatable. Multifunctional materials are constantly being developed, and will be a powerful addition to the complex antibacterial environment we face in the future. Further studies are needed regarding these materials.

At present, part of the antibacterial material model can only be used under laboratory with precision instruments and complex programs. The development thereof requires sustained effort from all researchers to achieve significant progress. Antibacterial material is an important consideration for long-term stability and the ability to maintain implant performance, and the commercialization of the antibacterial material to be used more frequently in the future is very important.
